# Optimized polymer-based glucose release in microtiter plates for small-scale *E. coli* fed-batch cultivations

**DOI:** 10.1186/s13036-020-00247-0

**Published:** 2020-08-27

**Authors:** Timm Keil, Barbara Dittrich, Clemens Lattermann, Jochen Büchs

**Affiliations:** 1 AVT - Biochemical Engineering, RWTH Aachen University, Forckenbeckstraße 51, 52074 Aachen, Germany; 2 DWI – Leibniz Institute for Interactive Materials, Forckenbeckstraße 50, 52074 Aachen, Germany; 3 Kuhner Shaker GmbH, Kaiserstraße 100, 52134 Herzogenrath, Germany

**Keywords:** Fed-batch, Microtiter plate, *Escherichia coli*, Screening, High-throughput, Bioprocess development, Overflow metabolism, Catabolite repression

## Abstract

**Background:**

Small-scale cultivation vessels, which allow fed-batch operation mode, become more and more important for fast and reliable early process development. Recently, the polymer-based feeding system was introduced to allow fed-batch conditions in microtiter plates. Maximum glucose release rates of 0.35 mg/h per well (48-well-plate) at 37 °C can be achieved with these plates, depending on the media properties. The fed-batch cultivation of fluorescent protein-expressing *E. coli* at oxygen transfer rate levels of 5 mmol/L/h proved to be superior compared to simple batch cultivations. However, literature suggests that higher glucose release rates than achieved with the currently available fed-batch microtiter plate are beneficial, especially for fast-growing microorganisms. During the fed-batch phase of the cultivation, a resulting oxygen transfer rate level of 28 mmol/L/h should be achieved.

**Results:**

Customization of the polymer matrix enabled a considerable increase in the glucose release rate of more than 250% to up to 0.90 mg/h per well. Therefore, the molecular weight of the prepolymer and the addition of a hydrophilic PDMS-PEG copolymer allowed for the individual adjustment of a targeted glucose release rate. The newly developed polymer matrix was additionally invariant to medium properties like the osmotic concentration or the pH-value. The glucose release rate of the optimized matrix was constant in various synthetic and complex media. Fed-batch cultivations of *E. coli* in microtiter plates with the optimized matrix revealed elevated oxygen transfer rates during the fed-batch phase of approximately 28 mmol/L/h. However, these increased glucose release rates resulted in a prolonged initial batch phase and oxygen limitations. The newly developed polymer-based feeding system provides options to manufacture individual feed rates in a range from 0.24–0.90 mg/h per well.

**Conclusions:**

The optimized polymer-based fed-batch microtiter plate allows higher reproducibility of fed-batch experiments since cultivation media properties have almost no influence on the release rate. The adjustment of individual feeding rates in a wide range supports the early process development for slow, average and fast-growing microorganisms in microtiter plates. The study underlines the importance of a detailed understanding of the metabolic behavior (through online monitoring techniques) to identify optimal feed rates.

## Background

The pivotal factors to maximize product yields in a given host construct are the choice of the best clone and optimized cultivation conditions [1]. Here, feeding strategies are essential to maximize product yields by keeping the organism in optimal metabolic conditions and to avoid overflow metabolism or catabolite repression.

The use of fed-batch cultivation conditions already in early process development steps leads to scalable results from small-scale to larger process stages [2–5]. There are several commercial available techniques enabling fed-batch cultivations in small-scale experiments. An overview of these techniques and commercially available systems are provided in Table 1. The Ambr 15 System manufactured by Sartorius AG uses special bioreactor vessels for cultivation and automated pipetting for feeding. This system provides flexible feeding profiles and almost all substrate solutions can be fed into the culture broth. However, pipette feeding always results in a liquid bolus feed and thus in fluctuating concentrations in the cultivation broth. Furthermore, the special bioreactor geometry does not allow standard microtiter plates as cultivation vessel. To apply feeding strategies in microtiter plates (MTPs) for early process development, there are only a few methods available [6]. One is the realization of feeding through micro-pumps, e.g. the BioLectorPro from m2p-labs GmbH [7, 8]. The Micro-matrix from Applikon Biotechnology uses a micro-injection valve as feeding technique. Those solutions enable precise process control (e.g. exponential feeding), but also require considerable investments into specialized equipment and the throughput is limited to only one MTP at a time. Other feeding techniques like the release of sugars by enzymatic degradation of polysaccharides directly into the culture broth are easy to use but very sensitive to environmental influences [9, 10]. This system is commercially available as Enpresso from Enpresso GmbH. The polymer-based fed-batch technique is an alternative method to allow fed-batch cultivations in MTPs. Here, the substrate is released from a silicone matrix [11], which is located at the bottom of each well. It does not require the acquisition of new equipment and is suitable for the release of other substances than sugars [12–15]. Although the sensitivity of the substrate release to environmental factors is reduced compared to the enzymatic release, the polymer-based feeding system also shows (reproducible) variations depending on various media properties like pH, osmotic concentration or ammonia content [14].

**Table 1:** An overview of techniques and commercially available systems that enable fed-batch cultivations in small-scale

	Ambr 15	Micro-Matrix	RoboLector	BioLector Pro	Enpresso	Standard FeedPlate
**Feeding technique**	Automated pipetting	Micro-injection valve	Automated pipetting	Micro-pumps	Degradation of polysaccharides	Continuous diffusion from polymer matrix
**Main advantage**	Flexible feeding profile and substrates	Flexible feeding profile and substrates	Flexible feeding profile and substrates	Flexible feeding profile	Simple, easy and cheap application in all scales. Continuous feed in standard MTP: No new device necessary	Simple, easy and cheap application; Continuous feed in standard MTP; No new device necessary
**Main drawback**	Significant investment, bolus feed	Significant investment, one plate per device	Significant investment, bolus feed	Significant investment	Release is strongly influenced by media and environmental properties	Release is influenced by media and environmental properties
**Applicable for MTPs**	No	Yes	Yes	Yes	Yes	Yes
**Parallel cultivations (per device)** ***N if the device/technic does not limit the number of parallel cultivations***	1 × 48	1 × 24	1 × 96	1 × 24	N × 96	N × 96

An *E. coli* fed-batch cultivation with the polymer-based feeding system considerably increased the productivity of a flavin mononucleotide binding fluorescence protein compared to the batch operation mode [3, 14]. The oxygen transfer rate (OTR) levels during the fed-batch phase in these cultivations were about 5 mmol/L/h. However, fed-batch studies in shake flasks showed that under fed-batch conditions OTR levels of up to 28 mmol/L/h are possible for *E. coli* cultivations [3]. Thus, *E. coli* is capable of utilizing higher glucose uptake rates than those supplied by the standard fed-batch MTP. Therefore, the full potential of *E. coli* as a production strain might not be exploited or a suboptimal strain might be selected for further process development in fed-batch experiments.

*E. coli* is a well-studied standard organism and provides beneficial properties for industrial processing like the ease of cultivation, low overall operating costs and high growth and product yields. A variety of efficient genetic tools are available [16], which make *E. coli* a preferred host for the production of recombinant proteins. Former obstacles of *E. coli*, for example, its lack of post-translational modifications, have been tackled by transferring the N-glycosylation systems of *Campylobacter jejuni* [17]. There are also intensive research efforts to overcome other crucial issues such as the production of proteins with molecular weights above 60 kDa, the formation of inclusion bodies for proteins with low solubility or codon bias for rare codons [18]. Thus, *E. coli* is and will remain one of the most important host systems for industrial biotechnology. In order to meet the requirements of industrially relevant, fast-growing microorganisms like *E. coli*, it is necessary to realize high feeding rates with the polymer-based release system.

The polymer-based feeding system is composed of a polymer matrix and embedded substrate particles. The polymer matrix consists of poly (dimethyl siloxane) (PDMS), a flexible, non-degradable polymer that is chemically inert and biocompatible [19]. It is used in medical implants, but also as a matrix for drug release systems, especially for the release of lipophilic drugs like steroids [20–23]. Although PDMS is hydrophobic, it exhibits high permeability to water vapor, which is a prerequisite for the osmotically driven release mechanism used in the here described system [24, 25]. If the polymer-based release system is immersed in aqueous solution, water vapor can penetrate the matrix and is bound in the embedded substrate particles. The resulting solution has high osmotic pressure and the gradient towards the surrounding medium is the driving force for further water ingress. This leads to an increase in the volume of the created pores and to the formation of cracks in the surrounding matrix, through which the substrate solution can escape into the surrounding medium [26, 27].

As mentioned before, the release characteristic is influenced by the media composition. Consistent with the release mechanism, all parameters that influence osmotic pressure, as well as solubility, may alter the release rate. Further determinants are the mechanical properties of the polymer matrix (e.g. the molecular weight of the prepolymer), the amount and the particle size of the embedded substrate crystals. Both factors will influence the fraction of water necessary to swell the pore until it cracks [28, 29]. Modifications in the molecular composition of the polymer matrix will also influence the release if e.g. the water permeability or the mechanical properties of the matrix are changed [30, 31].

In this study, the influence of the addition of a hydrophilic PDMS-PEG copolymer to the polymer-matrix and variations in molecular weights of the prepolymer are evaluated regarding the overall glucose release rate and the sensitivity of the release to environmental factors. Theoretically, the addition of the copolymer will increase the permeability of water through the matrix and thus increase the glucose release rate. The reduction of the molecular weight of the prepolymer might have a similar effect: The matrix becomes stiffer and thus simplifies the formation of cracks in the matrix. The glucose release rate of the adapted polymer-matrix is investigated in terms of medium composition and pH. Finally, MTPs equipped with a newly developed matrix with increased glucose release rates were used for fed-batch cultivations of *E. coli*. The overall goal is a fed-batch MTP that covers the demands of fast-growing microorganisms like e.g. *E. coli*, which cannot be provided by the existing plate.

## Material and methods

### Microtiter plates

In this study, 96-square-well (FeedPlate®, Part number: SMFP08002, Kuhner Shaker GmbH, Herzogenrath, Germany) and 48-round-well MTPs (MTP-R48-OFF, 60 m2p-labs GmbH, Beasweiler, Germany) were used. Fed-batch MTPs are produced by pouring the polymer matrix into each well of a MTP. The matrix, with embedded glucose crystals, hardens and provides the substrate reservoir. To achieve the desired release characteristics, the polymer matrix was adapted regarding the molecular weight of the used PDMS prepolymers (Gelest Inc. Morrisville, USA). The molecular weight of the prepolymer used in the standard matrix is approximately 20,000 g/mol, as given by the vendor. This value was confirmed by size-exclusion chromatography. For the new matrix, a prepolymer with a molecular weight of about 10,000 g/mol was used. Additionally, a PDMS-PEG copolymer with a PEG content of 50% (w/w) (gifted by Momentive Performance Materials GmbH, Leverkusen, Germany) was used as an additive to increase the hydrophilicity of the material. The molecular composition of the PDMS-PEG copolymer was confirmed by 1H-NMR. The glucose content in the *high release ++* matrix was 15% higher compared to the *high release* and the *high release +* matrix.

For all MTP glucose release experiments in 96-square-well and 48-round-well MTPs, “AeraSeal Film” (A9224, Sigma-Aldrich Chemie GmbH, Germany) sealings were used as a permeable sterile barrier [32]. “Polyolefin sealing foil” (900371-T, HJ-Bioanalytik GmbH, Erkelenz, Germany) was used for cultivations in 48-round-well MTPs.

### Microorganisms

*Escherichia coli* BL21 (DE3) was used as a prokaryotic model organism in this study.

### Media

To achieve comparability with previous works, the media compositions and procedures in this study are following those described in Keil et al. [14]. All components were purchased from either Carl Roth GmbH & Co. KG (Karlsruhe, Germany), Sigma Aldrich Chemie GmbH (Steinheim, Germany) or Merck KGaA (Darmstadt, Germany), if not otherwise stated.

In this work, all cultivations were performed in Wilms-MOPS medium. The base solution consists of 6.98 g/L (NH_4_)_2_SO_4_, 3 g/L K_2_HPO_4_, 2 g/L Na_2_SO_4_, 41.85 g/L (N-morpholino)-propane sulfonic acid (MOPS, 0.2 M) and 0.5 g/L MgSO_4_ × 7 H_2_O. The pH-value was adjusted to 7.5 with 10 mM NaOH solution. 1 mL/L of a sterile filtered 10 g/L thiamine chloride hydrochloride stock solution and 1 mL/L sterile filtered trace element solution were added to the base solution before each microbial cultivation. The trace element solution consists of 0.54 g/L ZnSO_4_ × 7 H_2_O, 0.48 g/L CuSO_4_ × 5 H_2_O, 0.3 g/L MnSO_4_ × H_2_O, 0.54 g/L CoCl_2_ × 6 H_2_O, 41.76 g/L FeCl_3_ × 6 H_2_O, 1.98 g/L CaCl_2_ × 2 H_2_O, 33.4 g/L Na_2_EDTA (Titriplex III, Merck, Darmstadt, Germany). No glucose was initially added to the medium for fed-batch cultivations. To create different osmotic concentrations in the medium for release experiments, no (= 0.284 Osmol/L) or 200 mM MOPS (=0.582 Osmol/L) were given to the medium. Additionally, different pH values (between 4.5 and 7.5) were investigated. MOPS buffer was replaced with equimolar 2-(N-morpholino)-ethane sulphonic acid (MES) buffer to analyze the glucose release into media with pH 5 and 4.5.

The base solution of Syn6-MES medium contains 1.0 g/L KH_2_PO_4_, 7.66 g/L (NH_4_)_2_SO_4_, 3.3 g/L KCl, 3.0 g/L MgSO_4_ × 7 H_2_O, 0.3 g/L NaCl, 27.3 g/L 2-(N-morpholino)-ethane sulphonic acid (MES). pH was adjusted to a value of 6.0 with 10 mM NaOH solution. No glucose was added to the medium.

Only the base solution of Wilms-MOPS and Syn6-MES medium was used for abiotic glucose release experiments. Except for the respective investigated parameter, all other parameters were not altered.

Phosphate buffered saline (PBS) tablets were employed (Part number: A9191, AppliChem GmbH, Darmstadt, Germany) to prepare PBS medium. Lysogeny broth (LB) medium, terrific broth (TB) medium and yeast extract peptone medium (YP) medium are complex media. LB medium consists of 10 g/L tryptone (Part number: 8952.4; Carl Roth GmbH & Co. KG, Karlsruhe, Germany), 5 g/L yeast extract (Part number: 2904.4; Carl Roth GmbH & Co. KG, Karlsruhe, Germany) and 5 g/L NaCl. TB medium consists of 12 g/L tryptone, 24 g/L yeast extract, 12.54 g/L K_2_HPO_4_ and 2.31 g/L KH_2_PO_4_. YP medium consists of 20 g/L tryptone and 10 g/L yeast extract. 0.2 g/L NaN_3_ was added to each medium in order to avoid the unintended growth of contaminants during all release experiments.

### Determination of glucose release kinetics

Glucose was spectrometrically analyzed with an enzymatic assay using glucose-6-phosphate-dehydrogenase and hexokinase based on the method proposed by Slein [33]. Each glucose data point corresponds to the mean value of three individually examined wells from which the standard deviation was calculated. The evaporation was determined gravimetrically and the data points were corrected accordingly. The release experiments were conducted in a cultivation hood (ISF1-X, Kuhner Shaker GmbH, Herzogenrath, Germany) at 37 °C and the MTPs were shaken at a frequency of 970 rpm at a shaking diameter of 3 mm. Each well was always filled with 1000 μL and the humidity inside the cultivation hood was kept constant at 80%.

### Cultivation procedure

For preculture, a 250 mL shake flask was filled with 10 mL Wilms-MOPS medium containing 20 g/L glucose. The preculture was inoculated with 500 μL from a cryo-stock (OD 10) and cultivated for 17 h at 30 °C on an orbital shaker with a shaking diameter of 50 mm at a shaking frequency of 300 rpm. Afterwards, the cells were centrifuged at 4000 rpm, 4 °C for 10 min in a Rotina 35 R (Andreas Hettich GmbH & Co. KG, Tüttlingen, Germany) and resuspended with fresh medium without glucose. The master mix of the main culture was inoculated with the resuspended cells to the desired starting OD (OD_start_). Thereafter, each well of a 48-square-well MTP was then filled with inoculated medium. The cultivations were performed at a temperature of 37 °C, a shaking frequency of 970 rpm, a shaking diameter of 3 mm and at a humidity of 80%. The initial pH was 7.5.

### Online measurements of the oxygen transfer rate in MTPs

In the 48-round-well fed-batch MTP, the OTRs were determined for each individual well with the Respiration Activity Monitoring System for MTPs (μRAMOS) technique described by Flitsch et al. [34].

### Offline sample analysis

pH was determined from the supernatant with HI2211 Basic pH / Redox / °C Meter (Hanna Instruments, Vöhringen, Germany). Osmotic concentration was measured with an Osmomat 3000 basic (Gonotec GmbH, Berlin, Germany). The device was three-point calibrated with DI-water and calibration standards (500 and 850 mOsmol/kg) prior to measurement.

## Results and discussion

### Optimizing matrix formulation

To develop a polymer-based feeding system with a high release of glucose for the cultivation of fast-growing microorganisms like *E. coli*, two approaches were chosen. First, the matrix polymer was cross-linked to a denser and thus stiffer network by introducing a prepolymer with lower molecular weight. Second, a hydrophilic PDMS-PEG copolymer was added to render the matrix more hydrophilic and by this increase the permeability of the matrix for water vapor.

**Fig. 1:**
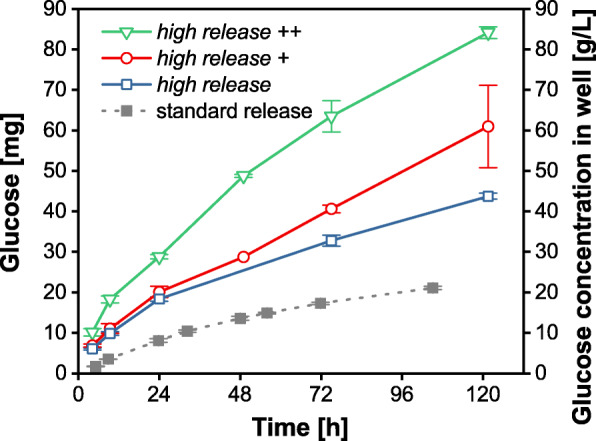
Glucose release per well of fed-batch MTPs into Wilms-MOPS medium with modified matrix composition. The left Y-axis represents the absolute amount of released glucose per well and the right Y-axis represents the measured glucose concentration in the experiment per well with V_L,48_ = 1000 μL/well. Grey data points correspond to the release of a 48-round-well fed-batch MTP with the standard matrix investigated in a previous study of our laboratory [14]. All data points represent the mean values of measurements of three individual wells. Error-bars indicate the respective standard deviation. General experimental conditions: 48-round-well MTP, temperature T = 37 °C, humidity = 80%, shaking frequency *n* = 970 rpm, shaking diameter d = 3 mm, V_L,48_ = 1000 μL/well. No initial glucose was provided in the medium. No microorganisms were inoculated, thus only the sterile medium was filled into the wells. To avoid unintended growth of contaminants, 0.2 g/L NaN_3_ was added to the medium

In Fig. 1, the amount of released glucose from 48-round-well MTPs is presented for four different polymer matrices. The grey data points represent the release of glucose from the standard matrix previously investigated in our laboratory and described and characterized by Keil et al. [14]. The standard matrix releases approximately 17.3 mg after 72 h. The blue data points depict the glucose release of the matrix formulation with a denser network and hydrophilic PDMS-PEG copolymer added (termed: *high release*). Compared to the standard matrix, the glucose release almost doubled (~ 30 mg after 72 h). The release characteristics show an increased glucose release rate in the first hours of the experiment (burst release). 24 h after the start of the experiment, the glucose release rate is almost constant resulting in a linear curve. The red data points represent the glucose release of a matrix with a denser network and with four times the amount of the hydrophilic copolymer (termed: *high release* +). Within the first 24 h, a burst release is observed as well. The further trend is similar to that of the *high release* matrix but at a slightly higher glucose level. 40 mg of glucose was released by the *high release +* matrix after 72 h. Data points in green depict the glucose release with 15% higher glucose content in the matrix, four times higher content of the hydrophilic copolymer in the matrix and denser network (termed: *high release* ++). The glucose release rate is further increased. After 72 h, almost 65 mg glucose was released from the matrix into each well.

These data indicate that a denser matrix network and the addition of hydrophilic PDMS-PEG copolymer provokes an increase in the glucose release rate. It is also evident that the glucose release rate is depending on the added amount of PDMS-PEG copolymer. The more hydrophilic the matrix, the easier it is for water molecules to diffuse through the matrix and dissolve glucose crystals. Additionally, the flux of water due to osmotic forces might increase through a more hydrophilic matrix as well. The data showed a considerable increase in the glucose release rate compared to the standard matrix. By altering the molecular weight of the prepolymer and adding a hydrophilic PDMS-PEG copolymer, the glucose release rate can be adjusted. A higher network density of the matrix, caused by a shorter molecular weight of the prepolymer, leads to a stiffer matrix. This results in less susceptibility of the matrix to swelling caused by imbibing water and thus to a quicker formation of cracks in the matrix. Consequently, the glucose release is faster in a stiffer matrix [25, 26, 29]. Besides, the more hydrophilic nature of the matrix results in a better permeability and a faster flux of water and substrate solution [30, 31, 35]. The additional increase of the embedded glucose content achieved a surplus in the release. One main task of this study was the increase of the glucose release rate to achieve higher OTR levels during fed-batch cultivations of *E. coli*. Thus, the matrix with increased glucose and hydrophilic polymer content (*high release ++*) was taken into account for further characterization.

### Evaluation of new matrix

**Fig. 2:**
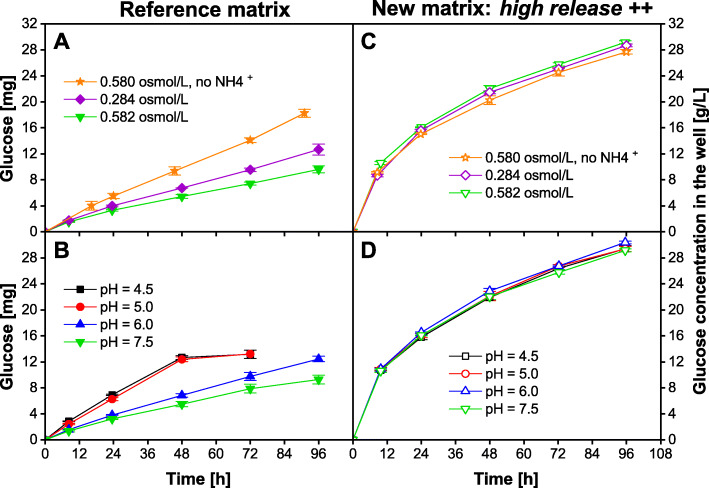
Glucose released per well of a standard 96-square-well fed-batch MTP (**a** + **b**) and the 96-square-well “*high release* ++” fed-batch MTP (**c** – **d**) into Wilms-MOPS medium. The left Y-axis represents the absolute amount of released glucose per well and the right Y-axis represents the measured glucose concentration in the experiment per well with V_L,96_ = 1000 μL/well. All data points are the mean values of measurements of three individual wells. Error-bars indicate the respective standard deviation. Except for the investigated property, all other properties were kept constant. Data in **a** + **b** were generated in our laboratory and previously published [14]. **a** + **c**) Influence of two osmotic concentrations on the total amount of glucose released. Additionally, in one approach ammonium was omitted. **b** + **d**) Influence of varying pH-values on the total amount of glucose released. For the pH-range from 6 to 8, 200 mM MOPS buffer was applied, for pH-values of 4.5 and 5, 200 mM MES buffer was applied. General experimental conditions: temperature T = 37 °C, humidity = 80%, shaking frequency *n* = 970 rpm, shaking diameter d = 3 mm, V_L,96_ = 1000 μL/well. No initial glucose was provided in the medium. No microorganisms were inoculated, thus only the sterile medium was filled into the wells. To avoid unintended growth of contaminants, 0.2 g/L NaN_3_ was added to the medium

The influence of medium properties on the release of the optimized matrix was tested and compared to the sensitivity of the standard matrix. It was already shown that for the standard matrix there is a predictable influence of media properties as depicted in the data set in Fig. 2 a and b. The data sets represent an extract of the glucose release data of the standard 96-square-deepwell fed-batch MTP generated in our laboratory [14]. In Fig. 2 a, the glucose release into standard Wilms-MOPS medium (Fig. 2 a: green triangles), as well as the release into adapted Wilms-MOPS medium with lower MOPS concentration and thus lower osmotic concentration (Fig. 2 a: purple diamonds) and into adapted Wilms-MOPS medium lacking ammonium salt (Fig. 2 a: orange stars) is depicted. The reason for reduced release rates with ammonium present in the medium could not be elucidated in detail, but a reaction of ammonium and glucose catalyzed by the Pt-catalyst present in the polymer matrix was hypothesized. Since the release data were gained with 96-square-deepwell MTPs, the performance of the optimized matrix (*high release* ++) is investigated in the same MTPs. The glucose release from the newly developed *high release ++* matrix is depicted in Fig. 2 c. All release experiments with this matrix show no considerable variations of the glucose release rate as a response to the osmotic concentration and the absence or presence of an ammonium salt.

In Fig. 2 b and c, the glucose release into Wilms-MOPS media with varying pH-values is shown for the standard (b) and the *high release ++* matrix (d). The glucose release rate of the standard matrix showed a strong dependency on the pH-value. At lower pH-values, the glucose release rate increases (Fig. 2 b). In contrast, the *high release ++* matrix showed a constantly high glucose release rate, independent of the pH-value (Fig. 2 d).

During the cultivation of *E. coli* strains, organic acids like acetate are typically produced due to overflow metabolism. These organic acids evoke a decrease of the pH-value in the medium. Thus, within the standard matrix, the glucose release rate would increase. This, in turn, would lead to a self-enhancing effect: higher glucose release rates cause reinforced production of acid, which itself increases the glucose release rate again. In the worst case, the glucose release rate exceeds the glucose uptake rate of the organism and glucose accumulates. In this case, glucose is not the limiting substrate anymore. The results from Fig. 2 verify that the glucose release rate of the *high-release ++* matrix is invariant to media composition and shifts of the pH-value, providing an additional operational benefit.

The OTR level of *E. coli* cultivations during the fed-batch phase with standard fed-batch MTPs in 96-square-well MTPs is about 5 mmol/L/h [14]. However, in membrane-based shake flasks, this strain was already cultured at a fed-batch OTR level of about 28 mmol/L/h [3]. Thus, the core objective of this study was an overall increase in the glucose release rate. The maximal amount of glucose released after 72 h was increased from 14 mg in the standard fed-batch MTP (Fig. 2 a: orange stars) to 25 mg with the new matrix (Fig. 2 c + d). Thus, the glucose release increased by 79% and, additionally, became less sensitive to the composition of media. The results from Fig. 2 show that the glucose release rate was noticeably increased for the new *high release ++* matrix.

**Fig. 3:**
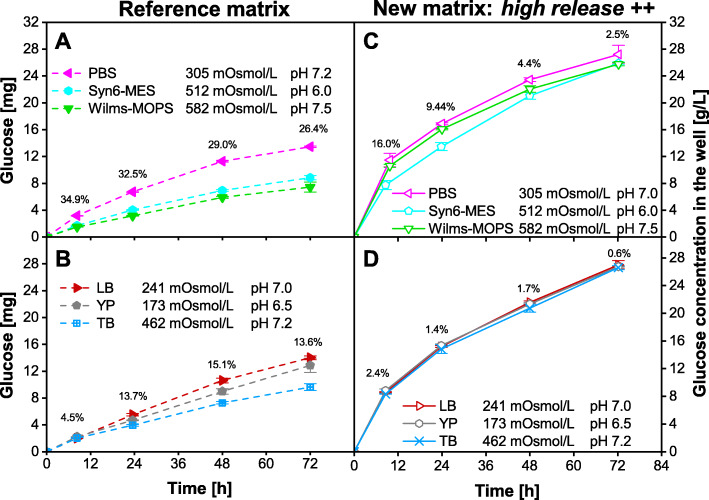
Glucose release of a standard 96-square-well fed-batch MTP (**a** + **b**) and of a *high release ++* fed-batch MTP (**c** + **d**) into commonly applied media. The left Y-axis represents the absolute amount of released glucose per well and the right Y-axis represents the measured glucose concentration in the experiment per well with V_L,96_ = 1000 μL/well. All data points represent the mean values of measurements of three individual wells. Error-bars indicate the respective standard deviation. The percentages reflect the deviation between the three different media per sample time. Data in **a** + **b** were generated in our laboratory and previously published [14]. **a** + **c**) Influence of the three synthetic media PBS, Syn6-MES and Wilms-MOPS. **b** + **d**) Influence of the three complex media LB, YP and TB. General experimental conditions: temperature T = 37 °C, humidity = 80%, shaking frequency *n* = 970 rpm, shaking diameter d = 3 mm, V_L,96_ = 1000 μL/well. No initial glucose was provided in the medium. No microorganisms were inoculated, thus only the sterile medium was filled into the wells. To avoid unintended growth of contaminants, 0.2 g/L NaN_3_ was added to the medium

In Fig. 3, the glucose release of the novel *high release ++* matrix into six commonly used media is depicted. In contrast to those tests discussed before, these media differ in several properties: their osmotic concentration, their initial pH-value, their component concentrations and their application area. Figure 3 a and b present the glucose release from the standard fed-batch MTP into three typical synthetic (a) and complex (b) media. In Fig. 3 c and d, the glucose release from the new *high release ++* matrix into synthetic and complex media is presented. The percentages reflect the deviation between the three different media per sample time. Except for the first two sample points in Fig. 3 c, the deviation among different media with the new *high-release ++* matrix is below 5%, whereas the deviation with the standard matrix is above 13% for almost all sample points of the standard matrix (Fig. 3 a and b). Even media with relatively low (YP) or high (Wilms-MOPS) osmotic concentration or different pH-values (Syn6-MES, pH-value 6; Wilms-MOPS, pH-value 7.5) show comparable glucose release rates (Fig. 3 c and d).

The optimized matrix enables constant glucose release rates that are independent of the applied media. By this, screening results become more reliable with the new polymer matrix. Up to now, changes in productivity or growth of microorganisms in fed-batch MTP cultivations could be the result of a successful medium optimization but also caused by shifting glucose release rates from the polymer matrix due to changing environmental conditions. With the newly developed polymer matrix, the influence of media properties on productivity and growth in small-scale fed-batch cultivations can now explicitly be investigated.

### Implementation of the new polymer matrix in biological experiments

**Fig. 4:**
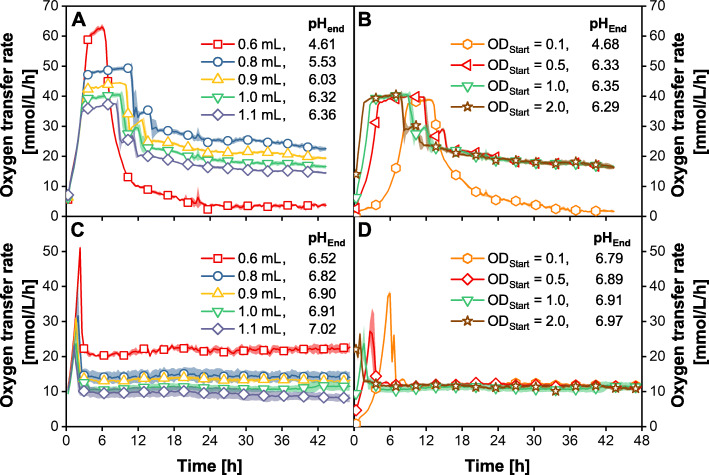
Cultivation data of *E. coli* BL21 (DE3) in 48-round-well *high release* ++ fed-batch MTPs. The OTR curves and the final pH-values at termination of *E. coli* BL21 (DE3) cultivations are presented. *High release ++* fed-batch MTP in (**c**) and (**d**) were washed prior to cultivation for 24 h. **a** + **c** Varying culture volume per well (0.6 mL – 1.1 mL). **b** + **d** Varying inoculation OD_Start_ (0.1–2.0). Culture conditions (unless otherwise stated): pH_0_ = 7.5, temperature T = 37 °C, humidity = 80%, shaking diameter d = 3 mm, shaking frequency *n* = 1000 rpm, culture volume V_L_ = 1000 μL/Well, OD_Start_ = 1.0, initial glucose concentration = 0 g/L. Shadow indicates standard deviation of six replicates. Symbols indicate every 10th data point

Microbial cultivations of *E. coli* with the newly developed fed-batch MTP were used to investigate the influence of increased glucose release rates in MTPs. Figure 4 a and b depict the OTR and the final pH of *E.coli* BL21 (DE3) cultivations in fed-batch MTPs prepared with the new *high-release ++* matrix. For these cultivations, 48-round-well MTPs were used with adapted shaking conditions (d = 3 mm; *n* = 1000 rpm) to allow online monitoring of the OTR by the μRAMOS device. In Fig. 4 a the initial culture volume was varied from 0.6 to 1.1 mL, the inoculum concentration remained constant at OD_start_ = 1.0. These culture volumes correspond to glucose release rates in the range from 0.75 g/L/h for 1.1 mL to 1.38 g/L/h for 0.6 mL filling volume, respectively (Additional file 1). For culture volumes higher than 0.8 mL, the OTR curves are typical for cultivations with fed-batch MTPs [14, 36, 37]. Initially, a batch phase of 3 h with exponential growth is observed due to accumulated glucose. Differently to OTR data with standard fed-batch MTPs [14], the OTR reaches a plateau after the exponential growth phase. The OTR of the occurring plateau is observed at lower values for increasing culture volumes. This plateau indicates an oxygen limitation due to a limited mass transfer of oxygen from the gas into the liquid phase. The OTR plateau is lowest for the highest culture volume since the volumetric mass transfer coefficient decreases with increasing culture volume. The specific maximum OTR values are in good agreement with typical maximum OTRs found under these cultivation conditions [38]. A smaller second peak following the oxygen limitation phase indicates the consumption of secondary metabolites [39]. After the initial batch phase and the consumption of secondary metabolites, a glucose-limited fed-batch phase is established (after 12–15 h, only for culture volumes ≥0.8 mL). The lower the culture volume, the higher the OTR level in the fed-batch phase. This is due to higher volumetric glucose release rates. OTR levels during the fed-batch phase of around 28 mmol/L/h could be achieved within a culture volume of 0.8 mL. This OTR level is in the same order of magnitude as in membrane-based fed-batch shake flasks and fermenter experiments of *E. coli* cultivations [3]. In contrast to a bolus feed with liquid handling robotics [40], a constant feed without generating a saw tooth profile is released with the polymer-based release technique. The final pH-value decreases with lower culture volumes, indicating a higher volumetric acid production or a higher volumetric ammonium consumption for lower culture volumes. This observation is reasonable since lower culture volumes result in higher volumetric glucose release rates. However, for all experiments, the final pH-value is below the optimal pH-value of *E. coli*, which is around 7.2 [41].

The experiment with 0.6 mL (red data points) in Fig. 4 a does not show a typical fed-batch OTR curve. Instead, after the initial exponential and oxygen-limited phase, the OTR decreases sharply to values below 10 mmol/L/h. This OTR pattern indicates a stop of the cultivation after about 6 h. The final pH-value of 4.61 suggests that the organisms acidify the media to such an extent that no further growth is possible.

The results in Fig. 4 a show that exceeding a specific glucose release rate (> 1.04 g/L/h) induces a premature stop of the cultivation if the glucose feed is started right from the beginning of the cultivation. Due to substantial glucose accumulation in the first phase of the experiment, the cultivations entail longer batch phases. These long batch phases at the beginning of the cultivation might suppress the desired benefits of fed-batch cultivation mode. Since the specific oxygen transfer coefficient is relatively small in 48-round-well MTPs, the cultures additionally suffer from oxygen limitations for several hours. This might lead to unfavorable growth and production conditions or the excessive formation of undesired acidic compounds.

In Fig. 4 b, the OTR of cultivations with varying starting ODs between 0.1 and 2.0 is presented. The culture volume was 1 mL per well, resulting in a constant glucose release rate of 0.83 g/L/h. The OTR of the cultivation with OD_start_ = 2.0 increased first, followed by OD_start_ = 1.0, then 0.5 and finally 0.1. The more biomass is present at the beginning of the cultivation, the faster the oxygen consumption increases. Eventually, all cultivations reach an OTR plateau of about 40 mmol/L/h, indicating an oxygen limitation (already discussed for the green curve in Fig. 4 a). Cultivations with OD_start_ ≥ 0.5 show an OTR pattern that indicates a fed-batch phase starting after 12–16 h with an OTR level of 20 mmol/L/h. Despite varying initial biomass concentrations, the cultures are in a similar state during this fed-batch phase. In all cases, an equal amount of glucose was released into each well at every point of time and the glucose release rate is equal as well. Thus, similar biomass concentrations and physiological state of the cells are expected [42].

The experiment with little initial biomass (OD_start_ = 0.1, yellow) sharply decreases after about 13 h and no typical subsequent fed-batch OTR level establishes. Again, the final pH-value of 4.68 indicates excessive acidification of the medium and a premature stop of the cultivation. The prolonged batch phase (overflow metabolism and oxygen limitation) due to little initial biomass causes too high organic acid production in relation to the applied buffer concentration. Thus, not only the glucose release rate but also the initial biomass concentration determines if a specific glucose release rate is too high for an aspired fed-batch cultivation.

In Fig. 4 a and b, cultivations with the new *high release ++* matrix reached OTR fed-batch levels of up to 28 mmol/L/h. However, some drawbacks appeared at these elevated glucose release rates, like long initial batch phases and oxygen limitation due to small volumetric mass transfer coefficients in 48-round-well MTPs. Additionally, the noticeable formation of secondary metabolites in the batch phase is undesired. This indicates that high glucose release rates are not necessarily beneficial for establishing optimal fed-batch cultivation conditions.

In Fig. 2 and Fig. 3, a relatively high initial glucose release rate is observed within the first 12 h of the experiment, which additionally encourages glucose accumulation right after the start of the cultivation and prolongs the batch phase in experiments. To circumvent the glucose release burst, each well of a fed-batch MTP with the new *high-release ++* matrix was filled with 1000 μL DI-water and incubated at standard cultivation conditions for 24 h prior to the cultivation (washing step). The cultivation directly started after the DI-water was removed by transferring inoculated medium into the wells. Figure 4 c and d display the OTR of *E. coli* BL21 (DE3) cultivations in washed fed-batch MTPs with the new *high release ++* matrix. In Fig. 4 c, the culture volume and in Fig. 4 d, the starting optical density (OD_start_) was varied. As for the cultivations with non-washed MTPs, different culture volumes in Fig. 4 c showed typical OTR profiles of fed-batch cultivations. However, major differences compared to the cultivations in Fig. 4 a are identifiable. The batch phase is shorter than 3 h for all experiments, and no experiment showed an oxygen limitation during the batch phase. This is due to less released and thus accumulated glucose, which needs to be consumed before fed-batch conditions are established. Before oxygen limitation could appear, the organism consumed the initially accumulated glucose. All experiments resulted in higher final pH-values between 6.5 and 7.0 compared to the unwashed fed-batch MTPs. Even more importantly, no cultivation was prematurely stopped due to excessive acidification. Fed-batch OTR levels of around 20 and 13 mmol/L/h were achieved for 0.6 and 0.8 mL culture volume, respectively. With increasing culture volume, the OTR fed-batch level accordingly decreases due to higher dilution of the released glucose.

**Fig. 5:**
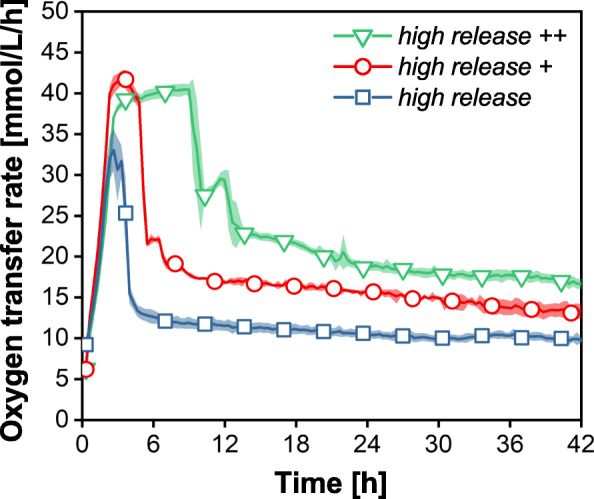
OTR data of *E. coli* BL21 (DE3) in different fed-batch MTPs (*high release* ++, *high release* +, *high release*). Culture conditions: pH_0_ = 7.5, temperature T = 37 °C, humidity = 80%, shaking frequency n = 1000 rpm, shaking diameter d = 3 mm, V_L_ = 1000 μL/Well, OD_Start_ = 1.0, initial glucose concentration = 0 g/L. Shadow indicates standard deviation of six replicates. Symbols indicate every 10th data point

In Fig. 4 d, OTR data of cultivations with washed fed-batch MTPs with varying initial OD_start_ are presented. In this case, the washing step has a similar impact on the cultivation. The batch phase is shortened and no oxygen limitation is observed. The maximum OTR fed-batch levels are lower (11–12 mmol/L/h) compared to those cultivations without a washing step (20–21 mmol/L/h). The final pH-values are in the range of 6.8 to 7.0. Consequently, washing the *high release ++* MTP is a suitable method to avoid high initial glucose release rates. However, washing the MTP before the application is an additional process step and might be an argument not to use the fed-batch MTP. An alternative way to adjust the glucose release rate already during the manufacturing process is the sophisticated combination of matrix components. In Fig. 1, the possibility to adjust the glucose release rate by varying the glucose content and adding hydrophilic PDMS-PEG copolymers was demonstrated. In Fig. 5, the OTR curves of cultivations with those three new matrices from Fig. 1 are presented. No washing step was applied prior to cultivation. The green curve represents the matrix with the highest release rate, which was thoroughly investigated before (*high release* ++). The red data represent a cultivation with reduced glucose content in the matrix (*high release* +). Compared to the *high release ++* system, the batch phase is already noticeably shortened, but the culture still runs into oxygen-limited conditions for 3 h. The fed-batch level is below the one of the *high release ++* matrix. The matrix with the additionally reduced hydrophilic copolymer (*high release*, blue data) showed an even shorter batch phase compared to the other cultivations. The fed-batch OTR level was further reduced. The less hydrophilic matrix leads to lower glucose release rates. The glucose and hydrophilic copolymer content determine the glucose release characteristics. A sophisticated combination of these parameters allows for the production of a tailored release system for specific applications.

The presented data show that the desired high glucose release rates specifically for fast-growing microorganisms were realized. In Table 2, a comparison of the newly developed fed-batch MTP with the existing fed-batch MTP is presented. The different MTPs are compared concerning the matrix components and the release characteristics. One major improvement of the newly developed fed-batch MTP is the higher glucose release rate and the resulting higher maximum OTR in the fed-batch phase. This allows fed-batch cultivations closer to the conditions prevailing in cultivations performed in a fermenter. All initially discussed drawbacks for the standard fed-batch MTP from Table 1 have been overcome with the newly developed fed-batch MTP. However, those high glucose release rates come along with long initial batch phases and potentially oxygen limitation. High initial starting ODs and high culture volumes per well shorten the duration of the batch phase. If oxygen-limited conditions need to be strictly avoided, smaller glucose release rates should be implemented.

**Table 2:** Comparison between the standard fed-batch MTP and the fed-batch MTP that was developed in this study

	Standard fed-batch MTP	Fed-batch MTP developed in this study
**Matrix components**		
Molecular weight of the prepolymer	20,000 g/mol	10,000 g/mol
Addition of hydrophilic co-polymer	No	Yes
**Release characteristics**		
Absolut glucose release	17.3 mg in 72 h	65 mg in 72 h
Maximum OTR during fed-batch phase of *E. coli* cultivations	5 mmol/L/h	28 mmol/L/h
Media components influencing the glucose release characteristics	Clear influence of pH, osmotic concentration, ammonium content	Almost no influence of media properties
Initial glucose burst	Almost not detectable	Significant

## Conclusion

For fast and reliable process development in small-scale cultivation vessels, the possibility to apply fed-batch conditions is required. The fed-batch MTP satisfies this demand. It allows simple and cheap feeding and a continuous substrate release without generating a saw tooth profile in the substrate concentration of the culture broth. It is applicable without significant investments, which is in contrast to other approaches that rely on micro-pumps, automated pipetting or micro-injection valves. Some fast-growing and industrially highly relevant microorganisms like *E. coli* require high glucose feed rates to manifest their full potential [3, 14]. Until now, such high glucose release rates could not be achieved by the polymer-based fed-batch MTP. To address these shortcomings, the release-determining polymer matrix of the fed-batch MTPs was refined to allow high and adjustable feed rates. By this, OTR fed-batch levels were increased from 5 mmol/L/h to 28 mmol/L/h. The adaptations of the polymer matrix considerably reduced the impact of medium properties like pH and osmotic concentration on the glucose release, as it is usually found for other easily applicable release techniques like the enzymatic degradation of polysaccharides or for the standard fed-batch MTP [10, 14]. This study revealed that excessive substrate release rates could result in a prolonged initial batch phase and oxygen limitation. This finding stresses the importance of a detailed understanding of the metabolic behavior of the applied cultivation system and underlines the need for the application of online monitoring systems to achieve this goal.

## Supplementary information

**Additional file 1.** Glucose release per well of fed-batch microtiter plate into Wilms-MOPS medium with the high release ++ MTP.

## Data Availability

The datasets used and/or analyzed during the current study are available from the corresponding author on reasonable request.

## Abbreviations

HPLC: High-performance liquid chromatography
LB: Lysogeny broth
OD: Optical density at 600 nm
OD_start_: Optical density at 600 nm at the beginning of a cultivation
Osmo: Osmotic concentration
OTR: Oxygen transfer rate
PBS: Phosphate buffered saline
TB: Terrific broth
YP: Yeast extract peptone
MTP: Microtiter plate
