# Supplementary material for: Optimized polymer-based glucose release in microtiter plates for small-scale E. coli fed-batch cultivations
Source: J Biol Eng. 2020 Aug 27;14:24. doi: 10.1186/s13036-020-00247-0 (PMC7457294; doi:10.1186/s13036-020-00247-0)
Supplement: Supplementary file 1 — Additional file 1. Glucose release per well of fed-batch microtiter plate into Wilms-MOPS medium with the high release ++ MTP. [file 13036_2020_247_MOESM1_ESM.pdf]

Additional files

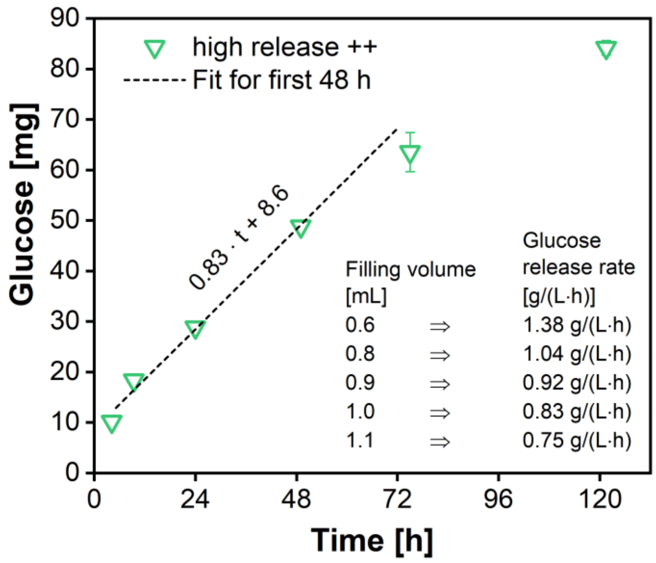

Additional file 1

Glucose release per well of fed-batch microtiter plate into Wilms-MOPS medium with the *high release* ++ MTP. All data points are the mean values of measurements of three individual wells. Error-bars indicate the respective standard deviation. The dashed black line indicates a linear fit of the glucose for the *high release* ++ MTP over the first 48 h. The slope of the fit describes the absolute glucose release rate. The (volumetric) glucose release rate for each filling volume is provided as table in the figure. General experimental conditions: 48-round-well MTP; temperature T = 37 °C, humidity = 80 %, shaking frequency n = 970 rpm, shaking diameter d = 3 mm, $V_{L,48}$ = 1000 µL/well. No initial glucose was provided in the medium. No biology was applied. To avoid unintended growth of contaminants, 0.2 g/L NaN_3 was added to the medium
